# Protective effects of mefunidone on ischemia-reperfusion injury/Folic acid-induced acute kidney injury

**DOI:** 10.3389/fphar.2022.1043945

**Published:** 2022-11-23

**Authors:** Jiajia Li, Yupeng Jiang, Qin Dai, Yue Yu, Xin Lv, Yan Zhang, Xiaohua Liao, Liyun Ao, Gaoyun Hu, Jie Meng, Zhangzhe Peng, Lijian Tao, Yanyun Xie

**Affiliations:** ^1^ Department of Nephrology, Xiangya Hospital, Central South University, Changsha, China; ^2^ Hunan Key Lab of Organ Fibrosis, Changsha, China; ^3^ National International Collaborative Research Center for Medical Metabolomics, Xiangya Hospital, Central South University, Changsha, China; ^4^ Department of Oncology, The Second Xiangya Hospital, Central South University, Changsha, China; ^5^ Department of Medicinal Chemistry, Xiangya School of Pharmaceutical Sciences, Central South University, Changsha, China; ^6^ Department of Pulmonary and Critical Care Medicine, The Third Xiangya Hospital, Central South University, Changsha, China

**Keywords:** renal ischemia-reperfusion injury, folic acid, acute kidney injury, chronic kidney disease, mefunidone

## Abstract

Renal ischemia-reperfusion injury (IRI) is one of the most common causes of acute kidney injury (AKI). It poses a significant threat to public health, and effective therapeutic drugs are lacking. Mefunidone (MFD) is a new pyridinone drug that exerts a significant protective effect on diabetic nephropathy and the unilateral ureteral obstruction (UUO) model in our previous study. However, the effects of mefunidone on ischemia-reperfusion injury-induced acute kidney injury remain unknown. In this study, we investigated the protective effect of mefunidone against ischemia-reperfusion injury-induced acute kidney injury and explored the underlying mechanism. These results revealed that mefunidone exerted a protective effect against ischemia-reperfusion injury-induced acute kidney injury. In an ischemia-reperfusion injury-induced acute kidney injury model, treatment with mefunidone significantly protected the kidney by relieving kidney tubular injury, suppressing oxidative stress, and inhibiting kidney tubular epithelial cell apoptosis. Furthermore, we found that mefunidone reduced mitochondrial damage, regulated mitochondrial-related Bax/bcl2/cleaved-caspase3 apoptotic protein expression, and protected mitochondrial electron transport chain complexes III and V levels both *in vivo* and *in vitro*, along with a protective effect on mitochondrial membrane potential *in vitro*. Given that folic acid (FA)-induced acute kidney injury is a classic model, we used this model to further validate the efficacy of mefunidone in acute kidney injury and obtained the same conclusion. Based on the above results, we conclude that mefunidone has potential protective and therapeutic effects in both ischemia-reperfusion injury- and folic acid-induced acute kidney injury.

## 1 Introduction

Acute kidney injury (AKI) is characterized by a rapid decline in kidney function over a short period, with an increase in serum creatinine (SCr) levels and/or a decrease in urine production. It poses a significant threat to public health, with high morbidity and mortality (Molema et al., 2022). AKI can be caused by trauma, sepsis, surgery, or nephrotoxic drugs (Levey and James, 2017). Renal ischemia-reperfusion injury (IRI) is a critical factor causing AKI. IRI always occurs after major surgeries such as kidney transplantation and cardiac surgery and is an inevitable consequence in multiple hospital settings (Sakai et al., 2019; Battistone et al., 2020). IRI is characterized by a short period of organ blood flow restriction followed by blood flow restoration, which initiates multiple cascades of destructive reactions (Kar et al., 2020). Due to the lack of effective treatment, AKI can gradually develop into chronic kidney disease (CKD), which is also easy to combine with other diseases, resulting in serious consequences (Martina et al., 2016; Rahbar Saadat et al., 2021). Despite a steady increase in the incidence of IRI-induced renal damage globally, strategies for effective prevention or management of kidney damage remain limited (Cao et al., 2020; James et al., 2020; Kellum et al., 2021).

In addition, high doses of folic acid (FA)-induced AKI, a classic AKI model, can cause direct toxicity to renal tubular epithelial cells or block renal tubules by forming crystals in the renal lumen (Gupta et al., 2012; Martin-Sanchez et al., 2018; Li et al., 2021a), thereby causing damage to the kidney by promoting oxidative stress and inflammation (Martin-Sanchez et al., 2017; Wang et al., 2021). Therefore, there is an urgent need to develop a method to protect the kidneys from AKI and prevent CKD caused by severe damage.

Mefunidone (MFD) [1-(4-((3-(4-methylpiperazin-1-yl) propyl) amino) benzyl)-5- (trifluoromethyl) pyridin-2(1H)-one] is a novel pyridinone drug with good absorption and low toxicity and is mainly distributed in the kidney (Han et al., 2019). Mefunidone exhibits an obvious anti-fibrotic effect in diabetic nephropathy and the unilateral ureteral obstruction (UUO) model (Liu et al., 2015; Zhang et al., 2016; Jiang et al., 2021). However, the protective effects of mefunidone against AKI remain unknown.

Hence, the study aimed to investigate the protective role of mefunidone in ameliorating IRI-induced AKI and further explore the underlying mechanism. We also used a folic acid (FA)-induced AKI mouse model to further validate the efficacy of mefunidone in AKI.

## 2 Materials and methods

### 2.1 Reagents and materials

Mefunidone (Lot No. 21062601) was synthesized by the Pharmaceutical Sciences Department, Central South University (Changsha, China). Folic acid (FA) (F8758) and sodium bicarbonate (V900182) were purchased from Sigma-Aldrich. Antibodies against proteins NGAL (ab216462) and collagen Ⅰ (ab270993) were purchased from Abcam. Antibodies against Bax (^#^2772), cleaved-caspase3 (^#^9664), and α-smooth muscle actin (α-SMA) (^#^19245) were purchased from Cell Signaling Technology. Antibodies against proteins bcl2 (^#^T40056) were purchased from Abcam. Antibodies against OxPhos (45–8,099) were purchased from Invitrogen. Antibodies against E-cadherin (20874-1-AP), vimentin (60330-1-Ig), α-tubulin (66031-1-Ig), and GAPDH (^#^60004-1-Ig) were purchased from Proteintech. A tissue reactive oxygen species assay kit (DHE) (^#^D7008) was purchased from Sigma-Aldrich. FITC Annexin V and PI staining kit (556547) was purchased from BD Pharmingen. Cell counting kit-8 (K1018) was purchased from APExBIO Technology LLC. The TMRM assay kit (T668) was purchased from Thermo Fisher Scientific. Blood urea nitrogen (BUN) (C013-2-1) and serum creatinine (SCr) (C011-2-1) kits were obtained from the Nanjing Jiancheng Bioengineering Institute. All the chemicals used in this study were of analytical grade.

### 2.2 Experimental animals and model

Male C57BL/6 mice, weighing 20–22 g (6–8 weeks old), were obtained from Silaike Laboratory (Shanghai, China). All animals were housed in a pathogen-free environment with a light/dark cycle of 12 h. All animals were fed an *ad libitum* diet and water, with adaptive feeding for at least 7 days. All animal experiments were conducted strictly in accordance with the standards of humane treatment established by the Laboratory Animal Sciences Association and Laboratory Animal Sciences Center at Central South University.

#### 2.2.1 Ischemia-reperfusion injury-induced acute kidney injury

Male C57BL/6 mice were randomly assigned to six groups with five mice per group: sham group, IRI operation group, and IRI operation with different doses of mefunidone. Mefunidone was dissolved in normal saline and administered to mice by gavage (50, 75, 100, and 125 mg/kg/day) 2 days before modeling. Anesthesia was administered to the mice on the day of modeling before bilateral renal pedicle clamping (18055-04, FST, Germany). After the bilateral kidney pedicles were exposed under the flank incisions, both kidneys were clamped for 30 min. Body temperature was maintained between 36.5°C and 37°C with a temperature-controlled heating device during the surgical procedure. After removal of the bilateral clamps, the abdomen was closed, and warm saline (1 ml) was injected into the abdominal cavity. The mice were euthanized 48 h after modeling, and the kidney tissues and serum samples were collected for further experiments.

#### 2.2.2 Folic acid-induced acute kidney injury

Male C57BL/6 mice were assigned to three groups randomly, consisting of sham (n = 6), FA injection (n = 6), and FA injection with mefunidone treatment (n = 6). The vehicle solvent for FA (250 mg/kg) was sodium bicarbonate (300 mM). Mefunidone was dissolved in normal saline and administered to mice by gavage (100 mg/kg/day) 2 days before modeling. All mice were treated with a single intraperitoneal injection of 250 mg/kg FA or vehicle solvent. The mice were euthanized 48 h after modeling, and the kidney tissues and serum samples were collected for further experiments.

#### 2.2.3 Ischemia-reperfusion injury-induced chronic kidney disease

Male C57BL/6 mice were assigned to three groups randomly, consisting of sham (n = 6), IRI (n = 6), and IRI with mefunidone treatment (n = 6). Mefunidone was dissolved in normal saline and administered to mice by gavage (100 mg/kg/day) 2 days before modeling. Anesthesia was administered to the mice on the day of modeling before left-lateral renal pedicle clamping (18055-04, FST, Germany). After the left lateral kidney pedicles were exposed to the flank incisions, the left kidney was clamped for 30 min. Body temperature was maintained between 36.5°C and 37°C with a temperature-controlled heating device during the surgical procedure. After removal of the left-lateral clamps, the abdomen was closed, and warm saline (1 ml) was injected into the abdominal cavity. The mice were euthanized 14 days after modeling, and the kidney tissues were collected for further experiments.

### 2.3 Cell culture and cell hypoxia/reoxygenation (H/R) model

Human proximal renal tubular epithelial cells (HK-2) were purchased from Procell Life Science&Technology Co., Ltd. (Wuhan, China) and cultured in DME/F12 medium (Gibco, United States) supplemented with 1% penicillin-streptomycin (Gibco, United States) and 10% fetal bovine serum (FBS, Gibco, United States) under 5% CO_2_ and 95% air atmosphere at 37°C. HK-2 cells were cultured in a medium containing CoCl2 (7791-13-1, Changsha Jingkang New Material Technology Co. LTD., China) without nutrients (serum-free, glucose-free) for 6 h. The medium was then replaced with DME/F12 with or without mefunidone for 12 h to induce the hypoxia/reoxygenation (H/R) model.

### 2.4 Assessment of renal function

Serum creatinine and blood urea nitrogen were detected according to the kit instructions (Nanjing Jiancheng Bioengineering Institute).

### 2.5 Histological staining and immunohistochemistry (IHC)

Paraffin-embedded kidney tissue sections (4 µm-thick) were stained with hematoxylin-eosin (HE) and/or Masson’s trichrome for pathological analysis. The percentage of cortical tubules was semi-quantitatively graded in a blinded manner, as reported previously (Jin et al., 2013) (Jiang et al., 2019). Collagen accumulation was measured using Masson’s trichrome staining, with blue collagen deposition as a positive signal. The percentage of positive areas in the entire field of vision (except for large vessels) was sorted by 0–4 points: 0, normal; 1, <25%; 2, 25%–50%; 3, 50%–75%; and 4, >75% (Lin et al., 2005; Fang et al., 2019).

The sliced kidney sections were dewaxed, rehydrated, and treated with H_2_O_2_ (3%) for 20 min, pepsin (0.4%) for 20 min, and then incubated with 5% bovine serum albumin in PBS for 30 min. The sections were incubated overnight with anti-NGAL, anti-collagen I, or anti-vimentin antibodies at 4°C. The samples were then washed with PBS and incubated with secondary antibodies (Abcam, Cambridge, United Kingdom).

### 2.6 Immunofluorescence

Dihydroethidium (DHE) staining was performed to detect the ROS levels. Cryosections of the kidney tissue (4 µm) were incubated in the dark for 30 min at 37°C for staining. A fluorescence microscope (Nikon, Tokyo, Japan) was used to test ethidium fluorescence with excitation/emission at 488/610 nm.

### 2.7 Electronic microscopy

Fixed kidney tissues (1 mm) were incubated overnight at 4°C with 2.5% glutaraldehyde, followed by chemical treatment with osmium tetroxide. The kidney tissues were subsequently rinsed, fixed, paraffin-embedded, and mitochondrial morphology was observed and recorded using transmission electron microscopy (TEM).

### 2.8 Quantitative real-time PCR examination

RNA was extracted from the kidney tissues using TRIzol reagent (Invitrogen). cDNA was synthesized from total RNA using the Real Master Mix (Bio-Rad Laboratories, CA, United States). mRNA expression was detected using SYBR Green assay (Takara Bio Inc., Kusatsu, Japan). β-actin was used to normalize the mRNA ratios, and the mean ± standard error of the mean (SEM) was used to represent the final data. The primer sequences used were as follows: mouse MnSOD: forward, 5′-CAG​ACC​TGC​CTT​ACG​ACT​ATG​G-3′; reverse, 5′-CTCGGTGGCGTTGAG ATTGTT-3′; mouse CAT: forward, 5′-CCT​ATT​GCC​GTT​CGA​TTC​TC-3′; reverse, 5′-CCC​ACA​AGA​TCC​CAG​TTA​CC-3′; and mouse β-actin: forward, 5′-CTGTCCCTG TATGCCTCTG-3′; reverse, 5′-TTG​ATG​TCA​CGC​ACG​ATT-3′.

### 2.9 Western blot analysis

Proteins from mouse kidney tissues were extracted in ice-cold 2 × SDS, and the protein concentration was detected using a BCA protein kit (#23225, Thermo, United States). Proteins were separated by 8%–15% SDS-PAGE and transferred to PVDF membranes (Millipore, MA, United States). The membranes were blocked in 5% skim milk prepare by TBST (150 mmol/L NaCl, pH 7.6, 0.05% Tween 20, and 20 mmol/L Tris-HCl) for 1 h at room temperature. The membranes were incubated with the primary antibody at 4 C overnight, followed by hybridization with HRP-conjugated secondary antibodies at room temperature for 1 h the next day. ECL western blotting reagents (32106, Thermo, United States) were used to visualize the results, and ImageJ was used for quantification.

### 2.10 TUNEL assay

An *in situ* cell death detection kit (11684795910, Roche, Switzerland) was used to evaluate the kidney tissue sections of 4 µm-thick treated with paraffin embedding, following the manufacturer’s protocols.

### 2.11 Cell viability

Effect of mefunidone on cell viability by Cell Counting Kit-8 (CCK-8). Briefly, HK-2 cells were seeded at a density of 4 × 10^3^ cells/well in 96-well plates. After overnight culture, the cells were incubated with different doses of mefunidone (0, 20, 40, 80, 120, 160 μg/ml) in 96-well plates for 24 h. HK-2 cells were then incubated with CCK-8 solution at 37°C for 2 h, and cell viability was determined by measuring the absorbance at 450 nm with a microplate reader.

### 2.12 Flow cytometry assay cell apoptosis

HK-2 cells were seeded in 6-well plates and divided into three groups: control, H/R, and H/R plus mefunidone. Hypoxia injury was induced by a medium containing CoCl_2_ without nutrients (serum-free, glucose-free) for 6 h; then, the medium was replaced with DME/F12 with or without mefunidone (80 μg/ml) for 12 h. HK-2 cells were collected in centrifuge tubes, according to the manufacturer’s instructions, and Annexin V and PI staining kit was used to assess cell apoptosis by flow cytometry.

### 2.13 Detection of mitochondrial membrane potential (MMP)

HK-2 cells were seeded in 6-well plates and divided into three groups: control, H/R, and H/R plus mefunidone. Hypoxic injury was induced using a medium containing CoCl_2_ without nutrients (serum-free, glucose-free). After 6 h, the medium was replaced by DME/F12 with or without mefunidone (80 μg/ml) for 12 h. Collecting HK-2 cells in centrifuge tubes, after adding TMRM reagent, mitochondrial membrane potential was monitored by flow cytometry.

### 2.14 Statistical analysis

All data are expressed as mean ± standard error of the mean (SEM). One-way ANOVA with post hoc analyses using the LSD method was used for comparisons among groups. Statistical significance was set at *p* < 0.05.

## 3 Results

### 3.1 Mefunidone ameliorated ischemia-reperfusion injury-induced acute kidney injury

**FIGURE 1:**
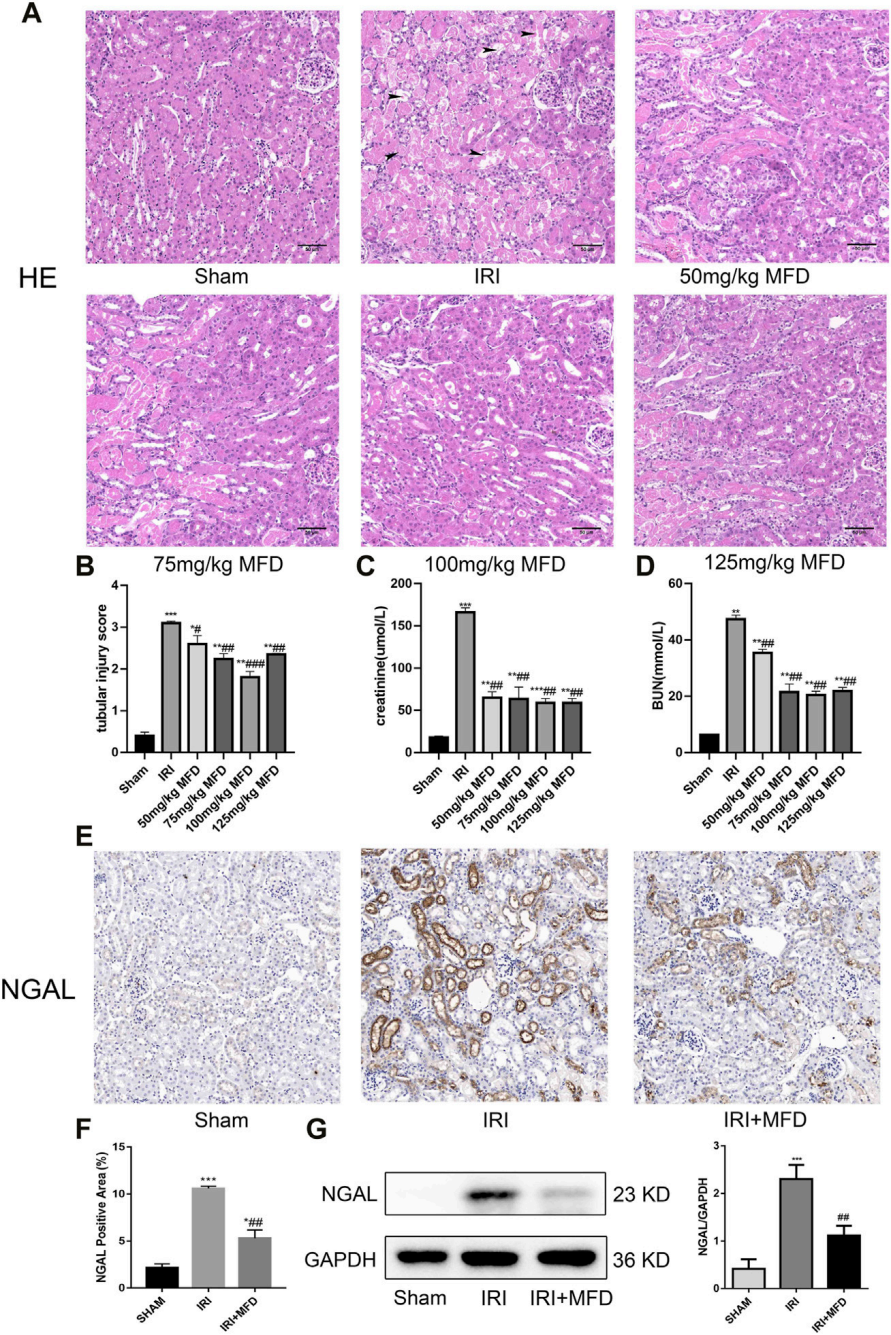
Mefunidone ameliorated IRI-induced AKI **(A)** HE staining showed protective effect of mefunidone at various doses of 50 mg/kg, 75 mg/kg, 100 mg/kg, 125 mg/kg on renal tubular injury on day 2 after IRI modeling (×200). arrows for renal tubular damage. **(B)** The tubular injury scores of HE staining for kidney damage. **(C)** Serum creatinine (SCr) levels of mefunidone at various doses of 50 mg/kg, 75 mg/kg, 100 mg/kg, 125 mg/kg on renal tubular injury on day 2 after IRI modeling. **(D)** Blood urea nitrogen (BUN) levels of mefunidone at various doses of 50 mg/kg, 75 mg/kg, 100 mg/kg, 125 mg/kg on renal tubular injury on day 2 after IRI modeling. **(E,F)** Histological images of immunohistochemical staining with NGAL and evaluation of NGAL positive area in each group on day 2 after IRI modeling (×200). Mefunidone: 100 mg/kg. **(G)** Western blot analysis and quantitative data of NGAL in each group on day 2 after IRI modeling. Mefunidone: 100 mg/kg. Data represent mean ± SEM (*n* = 3–5). *p < 0.05, vs. Sham group; **p < 0.01, vs. Sham group; ***p < 0.001, vs. Sham group; #*p* < 0.05, vs. IRI group; ##*p* < 0.01, vs. IRI group; ###*p* < 0.001, vs. IRI group.

The effect of mefunidone on IRI-induced AKI was determined by HE staining of kidney tissue sections (Figures 1A,B). Compared to the sham group, the IRI-treated group showed obvious kidney tubular injury, including proximal tubular brush border loss, tubular lysis, and cast formation. Mefunidone partially protected the tubular epithelium from proximal tubular brush border loss, tubular lysis, and cast formation, with remarkable effects at a concentration of 100 mg/kg/day. Compared with the sham group, the IRI-treated group showed significant renal dysfunction, and the mefunidone treatment group showed partially improved renal function (Figures 1C,D). These results indicated that different doses of mefunidone could attenuate renal IRI to varying degrees, and the most obvious effect was observed at 100 mg/kg/day. Therefore, a dose of 100 mg/kg/day was selected for all the subsequent experiments. These results were also supported by the decreased expression of NGAL observed using IHC and western blotting in the mefunidone-treated group (Figures 1E–G).

In this study, we explored the effects of long-term ischemia on fibrosis in IRI. We assessed the effects of mefunidone on day 14 after IRI modeling. Compared with the IRI-treated group, treatment with mefunidone significantly improved renal tubular injury and renal fibrosis in CKD induced by IRI, as shown in Supplementary Figure S1.

### 3.2 Mefunidone reduced ischemia-reperfusion injury-induced oxidative stress accumulation

**FIGURE 2:**
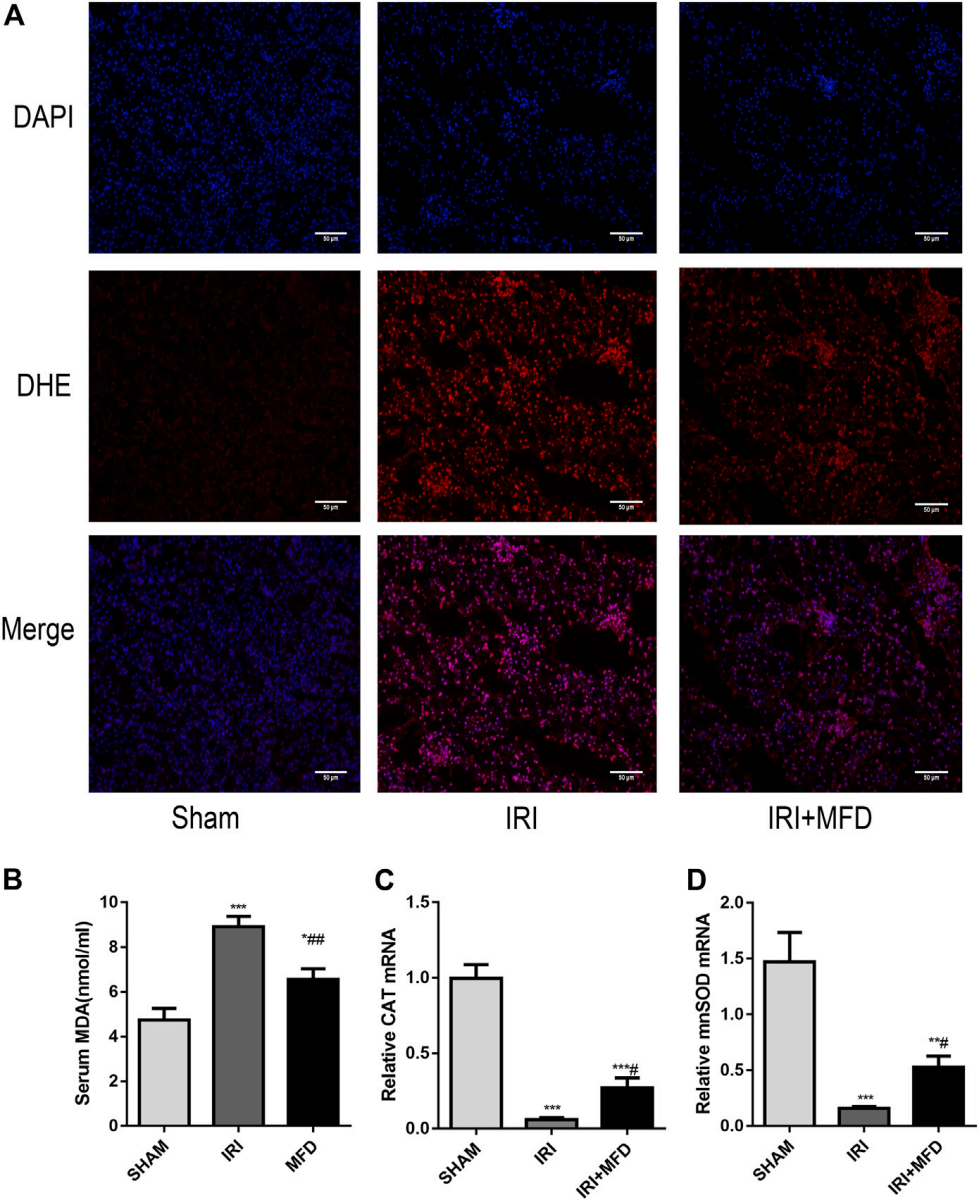
Mefunidone reduced IRI-induced oxidative stress accumulation. **(A)** DHE immunofluorescence staining for ROS detection in the renal tissues on day 2 after IRI modeling (×200). **(B)** Renal MDA level in the renal tissues on day 2 after IRI modeling. **(C)** RT-qPCR of CAT in the renal tissues on day 2 after IRI modeling. **(D)** RT-qPCR of mnSOD in the renal tissues on day 2 after IRI modeling. Mefunidone: 100 mg/kg. Data represent mean ± SEM (n = 3–5). *p < 0.05, vs. Sham group; **p < 0.01, vs. Sham group; ***p < 0.001, vs. Sham group; ^#^ *p* < 0.05, vs. IRI group; ^##^ *p* < 0.01, vs. IRI group.

Consistent with renal tubular damage, ROS accumulation was observed in the kidneys after IRI injury, and mefunidone treatment noticeably reduced ROS accumulation (Figure 2A). Mefunidone treatment also significantly decreased IRI-induced levels of serum MDA, a marker of lipid peroxidation (Chen et al., 2019a; Li et al., 2019a) (Figure 2B). In addition, the mRNA expression of catalase (CAT) and manganese superoxide dismutase (mnSOD) was elevated in the IRI-treated group, and treatment with mefunidone reversed these trends (Figures 2C,D).

### 3.3 Mefunidone abated renal apoptosis

**FIGURE 3:**
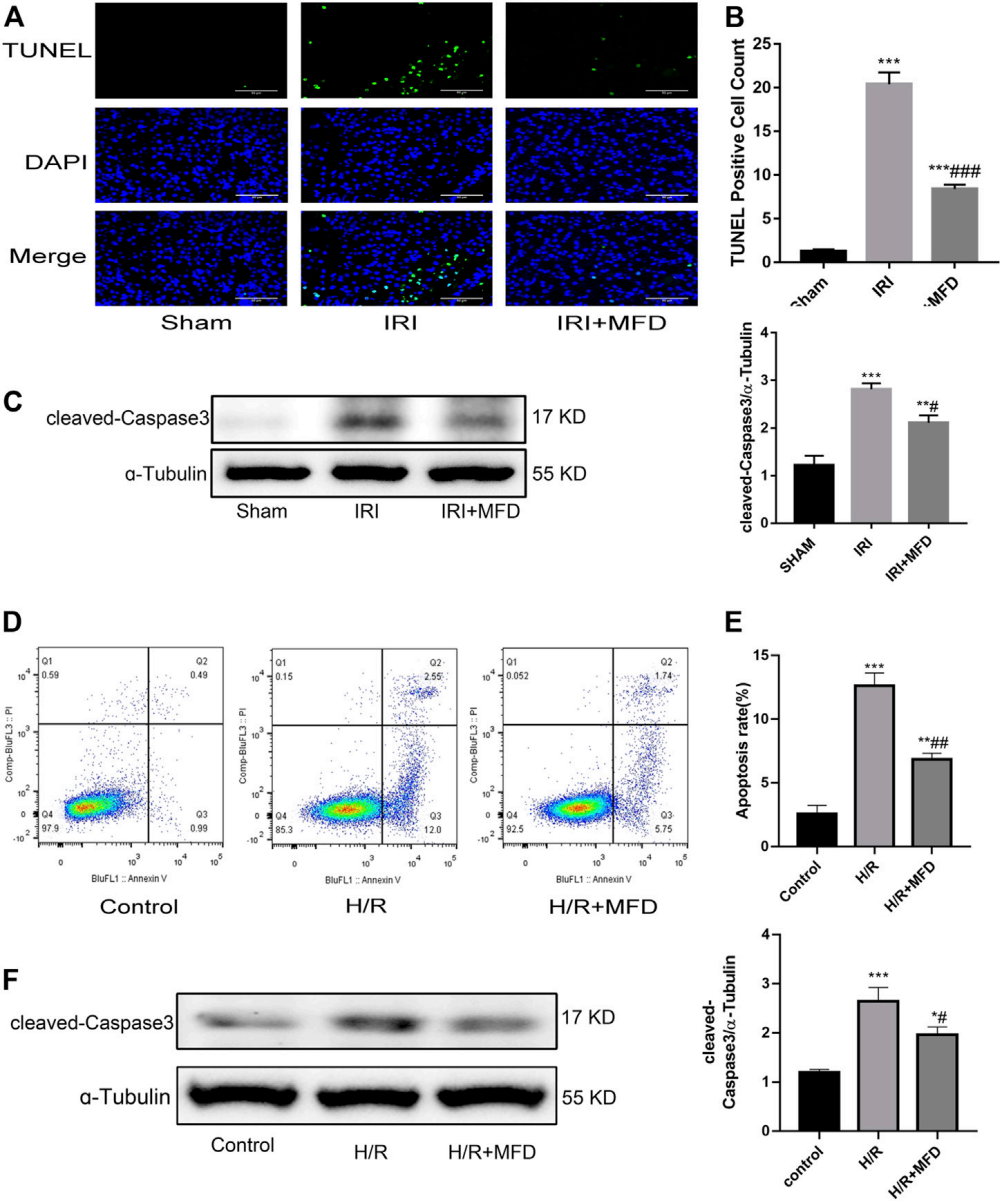
Mefunidone abated renal apoptosis **(A)** TUNEL staining in the renal tissues on day 2 after IRI modeling (×400). **(B)** Quantification of TUNEL positive cell count per scope. **(C)** Western blot analysis and quantitative data of cleaved-caspase3 in the renal tissues on day 2 after IRI modeling. **(D,E)** Flow cytometry and quantitative comparison were applied to detect apoptosis of HK-2 cells in H/R model with or without mefunidone. **(F)** Western blot analysis and quantitative data of cleaved-caspase3 in the H/R model of HK-2 cells with or without mefunidone. Dose of mefunidone in mice: 100 mg/kg; Dose of mefunidone in HK-2 cells: 80 μg/ml. Data represent mean ± SEM (n = 3–5). *p < 0.05, vs*.* Sham group; **p < 0.01, vs*.* Sham group; ***p < 0.001, vs. Sham group; ^#^ *p* < 0.05, vs. Modeling group; ^##^ *p* < 0.01, vs. Modeling group; ^###^ *p* < 0.001, vs. Modeling group.

Previous studies have shown that apoptosis of renal tubular epithelial cells is involved in the pathological process of renal injury induced by IRI (Priante et al., 2019; Ma et al., 2020; Pan et al., 2020). TUNEL staining was performed to investigate whether mefunidone can improve IRI-mediated renal apoptosis. The results revealed that mefunidone treatment significantly attenuated renal apoptosis following IRI-induced AKI (Figures 3A,B). Subsequently, we examined the expression of cleaved-caspase3, which is an apoptotic marker. We observed an increase in cleaved-caspase3 expression in the IRI-treated group, whereas mefunidone treatment decreased the expression of cleaved-caspase3 (Figure 3C). Then we verified the therapeutic effect of mefunidone *in vitro*. First, we used CCK-8 to determine the optimal drug concentration of mefunidone for 24 h for HK-2 cell viability. As shown in Supplementary Figure S2, 80 μg/ml mefunidone showed no obvious toxicity to HK-2 cells. We selected a concentration of 80 μg/ml mefunidone for the following experiments. We used flow cytometry to detect apoptosis of HK-2 cells, consistent with animal experiments. The apoptosis rate of HK-2 cells in the H/R group was relatively high, and the number of apoptotic HK-2 cells decreased significantly after treatment with mefunidone (Figures 3D,E). We also detected the cleaved-caspase3 expression in the H/R model of HK-2 cells. The cleaved-caspase3 expression was increased in the H/R group, and mefunidone downregulated the expression of cleaved-caspase3 (Figure 3F).

### 3.4 Mefunidone protected IRI-induced mitochondrial damage and complex Ⅲ and Ⅴ of mitochondrial electron transport chain complex

**FIGURE 4:**
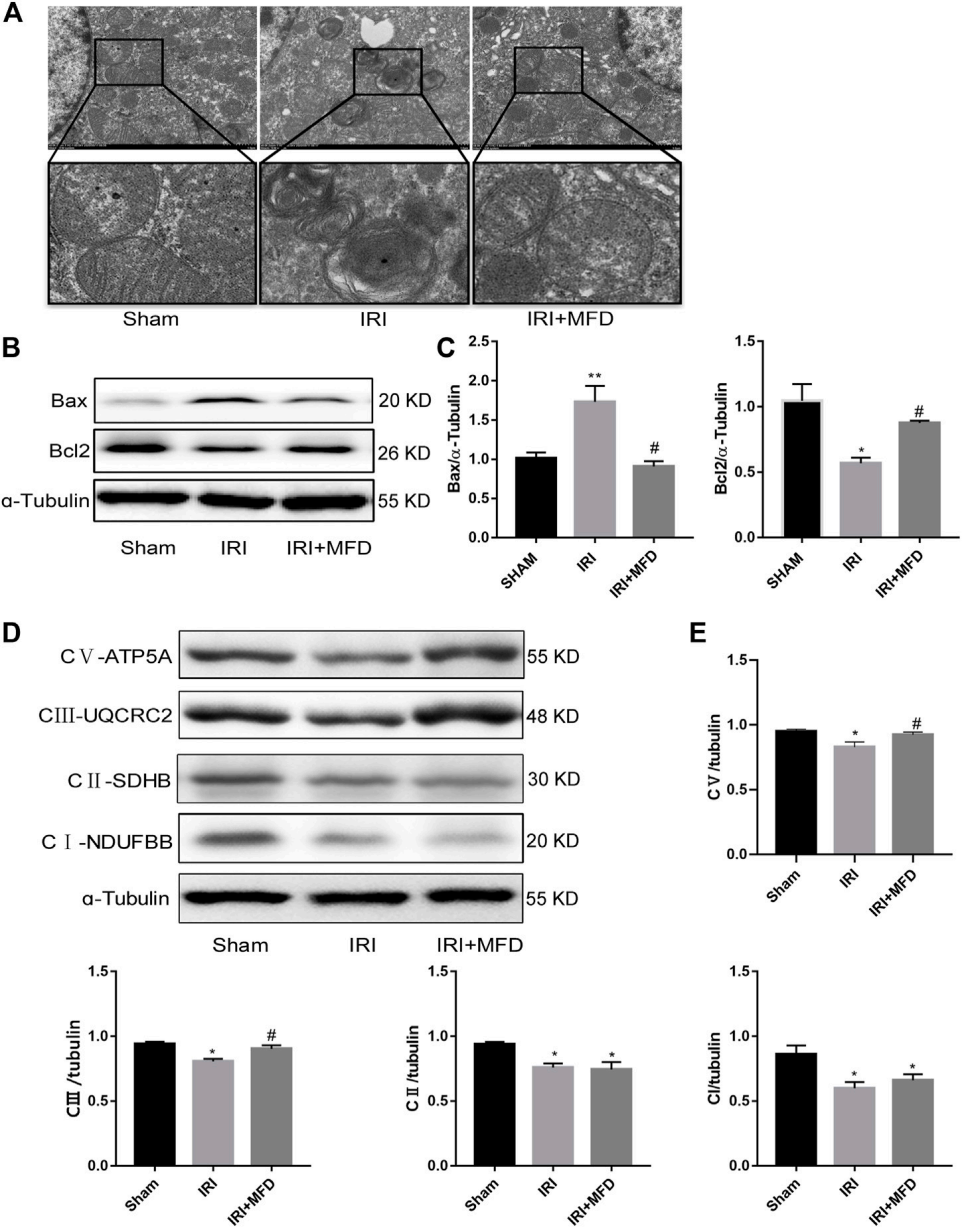
Mefunidone protected IRI-induced mitochondrial damage and complex Ⅲ and Ⅴ of mitochondrial electron transport chain complex **(A)** Electron microscope analysis of mitochondria in the renal tissues on day 2 after IRI modeling (×8,000). **(B,C)** Western blot analysis and quantitative data of bax and bcl2 in the renal tissues on day 2 after IRI modeling. **(D,E)** Western blot analysis and quantitative data of mitochondrial electron transport chain complex in the renal tissues on day 2 after IRI modeling. Mefunidone: 100 mg/kg. Data represent mean ± SEM (n = 3). *p < 0.05, vs*.* Sham group; **p < 0.01, vs*.* Sham group; ^#^ *p* < 0.05, vs. IRI group.

We used TEM to detect the mitochondria, and the results clearly showed the mitochondrial cristae and intact mitochondrial membrane in the sham group. Mitochondrial swelling and vacuolation appeared in the IRI-treated group, although the mitochondrial integrity status remained unchanged, and mitochondrial damage partially improved after mefunidone treatment (Figure 4A). Since we observed that mefunidone could improve mitochondrial damage and that the mitochondrial pathway is the most important apoptotic mechanism in AKI, we examined whether mefunidone treatment abated IRI-mediated renal apoptosis by inhibiting the expression of mitochondrial-related pro-apoptotic proteins. Western blot analysis confirmed that in the IRI-treated group, Bax expression increased while bcl2 expression decreased. Mefunidone treatment reduced Bax expression and increased bcl2 expression (Figures 4B,C). Moreover, we evaluated mitochondrial electron transport chain protein expression and found that complex I, complex II, complex III, and complex V protein expression in the IRI-treated group decreased significantly compared with those in the sham group, and mefunidone partially recovered complex III and complex V, but not the downregulation of complex I and complex II (Figures 4D,E).

### 3.5 Mefunidone reduced H/R-induced mitochondrial damage and complex Ⅲ and V of mitochondrial electron transport chain complex in HK-2 cells

**FIGURE 5:**
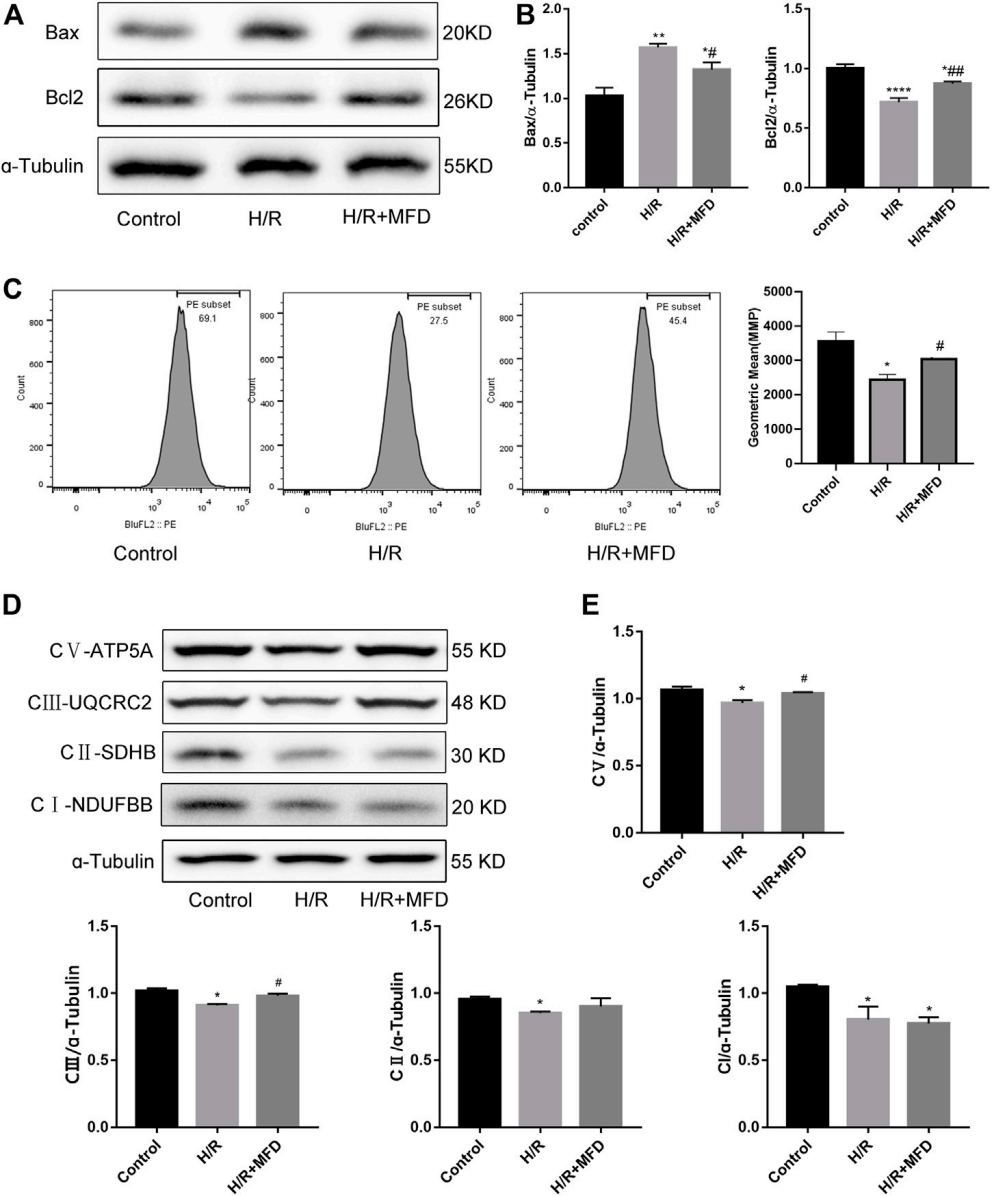
Mefunidone reduced H/R-induced mitochondrial damage and complex Ⅲ and Ⅴ of mitochondrial electron transport chain complex in HK-2 cells **(A,B)** Western blot analysis and quantitative data of bax and bcl2 in the H/R model of HK-2 cells with or without mefunidone. **(C)** Flow cytometry and quantitative comparison were applied to test MMP of HK-2 cells in the H/R model of HK-2 cells with or without mefunidone. **(D,E)** Western blot analysis and quantitative data of mitochondrial electron transport chain complex in the H/R model of HK-2 cells with or without mefunidone. Mefunidone: 80 μg/ml. Data represent mean ± SEM (n = 3–5). *p < 0.05, vs. Control group; **p < 0.01, vs. Control group; ***p < 0.001, vs. Control group; ****p < 0.0001, vs. Control group; ^#^ *p* < 0.05, vs. H/R model group; ^##^ *p* < 0.01, vs. H/R model group.

To verify the protective effect of mefunidone on mitochondria *in vitro*, we measured the expression of mitochondria-related apoptotic proteins (Figures 5A,B). We found that mefunidone downregulated the expression of the pro-apoptosis-related protein Bax while upregulating bcl2 expression, which is in accordance with our observations *in vivo*. Subsequently, we detected changes in MMP and the mitochondrial electron transport chain complex expression in HK-2 cells. As shown in Figure 5C, mefunidone treatment significantly improved the MMP decline caused by the H/R model. Compared with the control group, complex I, complex II, complex III, and complex V protein expression decreased significantly in the H/R group, and mefunidone partially recovered complex III and complex V but did not downregulate complex I and complex II (Figures 5D,E).

### 3.6 Mefunidone ameliorated folic acidinduced acute kidney injury

**FIGURE 6:**
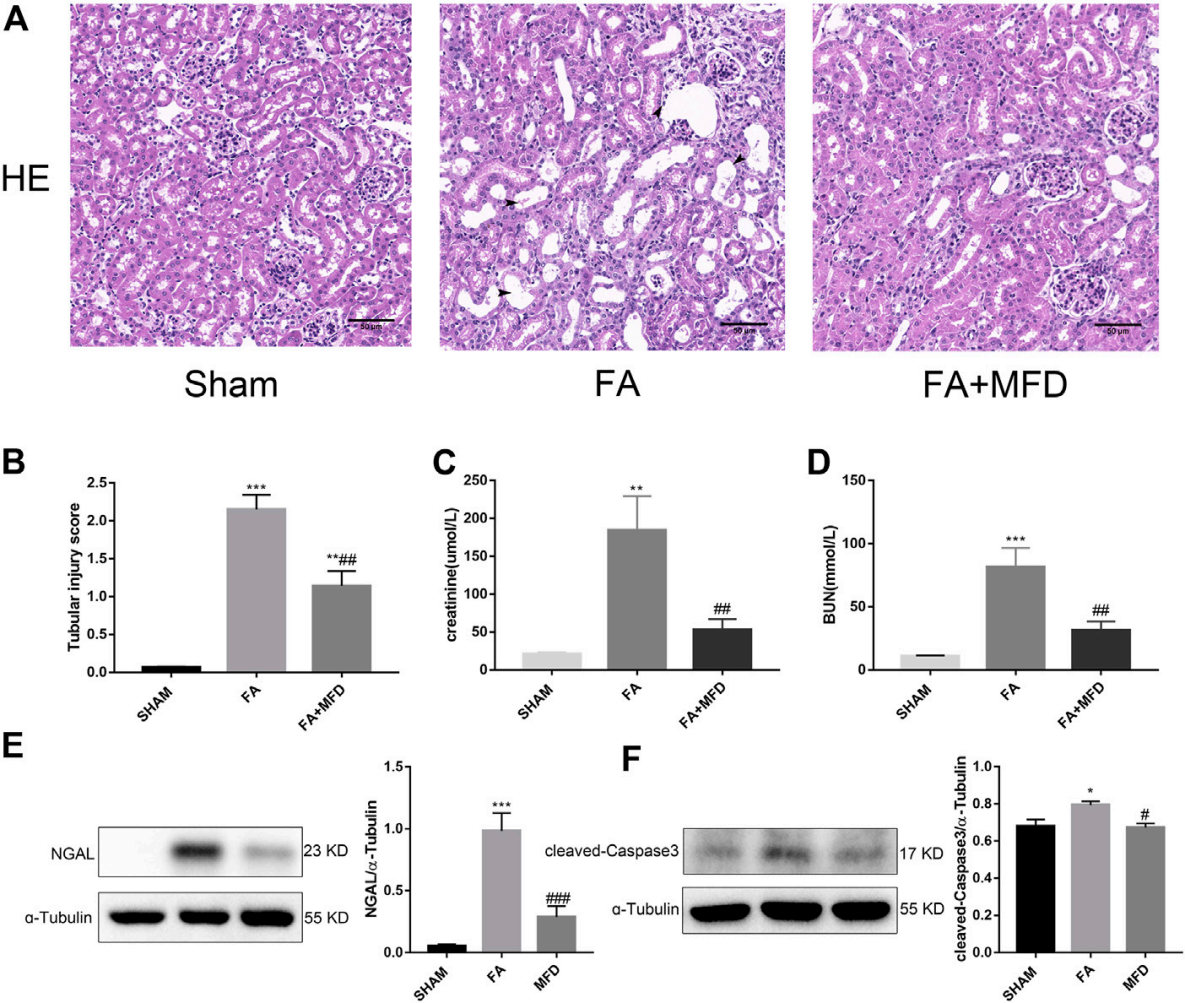
Mefunidone ameliorated FA-induced AKI **(A)** HE staining showed protective effect of mefunidone on renal tubular injury on day 2 after FA modeling. arrows for renal tubular damage. (×200). **(B)** The tubular injury scores of HE staining for kidney damage. **(C)** Serum creatinine (SCr) levels on renal tubular injury on day 2 after FA modeling. **(D)** Blood urea nitrogen (BUN) levels on renal tubular injury on day 2 after FA modeling. **(E)** Western blot analysis and quantitative data of NGAL in each group on day 2 after FA modeling. **(F)** Western blot analysis and quantitative data of cleaved-caspase3 in each group on day 2 after FA modeling. Mefunidone: 100 mg/kg. Data represent mean ± SEM (n = 3–6). *p < 0.05, vs. Sham group; **p < 0.01, vs. Sham group; ***p < 0.001, vs. Sham group; ^#^ *p* < 0.05, vs. FA group; ^##^ *p* < 0.01, vs. FA group.

To explore whether mefunidone also protects against FA-induced AKI, HE staining was applied to observe and evaluate the severity of tubular injury induced by FA in AKI. Compared to the sham group, the model group receiving FA injection showed obvious tubular dilatation, brush border deletion, cell debris, and vacuolization. The above situation significantly improved after mefunidone treatment (Figures 6A,B). Analysis of renal function revealed that serum creatinine and blood urea nitrogen in FA treated group were significantly higher in the FA-treated group than in the sham group. The mefunidone treatment group partially reduced the serum creatinine and blood urea nitrogen levels to maintain renal function (Figures 6C,D). These results were also supported by the decreased expression of NGAL in the mefunidone-treated group, as determined by western blotting (Figure 6E). Similarly, to determine whether mefunidone could also inhibit apoptosis in the FA-induced AKI model, we examined the expression of the apoptosis marker cleaved-caspase3. We observed an increase in cleaved-caspase3 expression in the FA group, whereas mefunidone treatment decreased the expression of cleaved-caspase3 (Figure 6F).

## 4 Discussion

AKI is a serious health risk owing to its high incidence, tendency to combine with other diseases, and poor prognosis (Martina et al., 2016). AKI occurs in approximately 13 million people worldwide each year, of which approximately 1.7 million die from AKI (Mehta et al., 2015). We know that IRI is a key contributor to AKI, and usually occurs after major surgery such as kidney transplantation and cardiac surgery, which is predictable in clinical (Sakai et al., 2019; Battistone et al., 2020; Bellini et al., 2021). Based on the clinical background, we can reduce renal damage by prophylactic administration of drugs before the onset of renal injury is anticipated.

**FIGURE 7:**
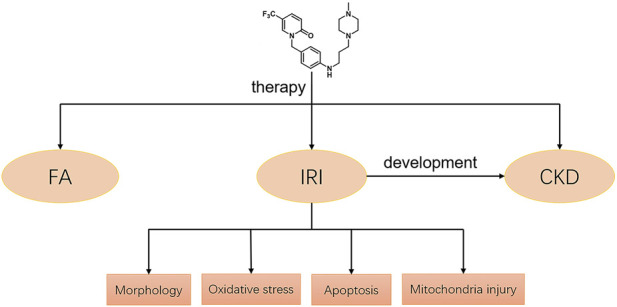
Potential mechanisms of mefunidone for the treatment of IRI/FA-induced AKI. Mefunidone may improve IRI-induced AKI by inhibiting oxidative stress, reducing apoptosis and protecting mitochondria.

Our study demonstrated that a novel pyridinone drug, mefunidone, effectively ameliorated IRI-induced AKI by suppressing oxidative stress, maintaining mitochondrial structure, inhibiting kidney tubular epithelial cell apoptosis by regulating mitochondrial-related Bax/bcl2/cleaved-caspase3 apoptotic protein expression, and protecting mitochondrial electron transport chain complex III and complex Ⅴ levels. In addition, mefunidone significantly improved renal tubular injury and fibrosis in CKD induced by IRI. Simultaneously, we further elucidated the efficacy of mefunidone in AKI in an FA-induced AKI model. The continuous effects of mefunidone may decrease the life-threatening condition of AKI and promote long-term renal benefits. These results suggest a promising future for the use of mefunidone in the preventive treatment of IRI and FA-induced AKI (Figure 7).

In this study, we found that mefunidone had a protective effect against IRI-induced AKI. HE staining revealed that mefunidone protected the tubular epithelium from tubular lysis, brush border loss, cast formation, and improved kidney tubular injury scores. Similarly, this conclusion was further validated in renal function tests, which showed that mefunidone significantly reduced serum creatinine and blood urea nitrogen levels. Furthermore, we used IHC and western blotting to detect the expression of NGAL, a renal injury marker (Makris and Kafkas, 2012; Yu et al., 2018), and the results showed that mefunidone treatment could reduce the expression of NGAL in the IRI-induced AKI model. Thus, we conclude that mefunidone has a protective effect against IRI-induced AKI. Subsequently, we explored the therapeutic mechanism of mefunidone in IRI-induced AKI.

The ischemic kidney produces ROS during the reperfusion phase and triggers a series of deleterious cellular reactions, leading to endothelial dysfunction, cell death, and ultimately, organ failure (Nezu et al., 2017; Wu et al., 2019). Thus, numerous studies have reported potential effective improvements in IRI-induced AKI *via* anti-oxidant therapy (Liu et al., 2019; Rossi et al., 2019; Zhao et al., 2021). In this study, the detection of ROS production by DHE staining and mefunidone treatment significantly reduced the fluorescence intensity of DHE. The depletion of the anti-oxidant enzymes CAT and mnSOD in IRI is closely associated with oxidative damage (Han et al., 2017; Jiang et al., 2019). Mefunidone treatment sharply elevated the expression of these anti-oxidant enzymes. Furthermore, excessive oxidative damage causes not only direct damage but also induces further lipid peroxidation (Schleicher and Dahmen, 2018; Cui et al., 2021). Mefunidone also reduced MDA levels as a marker of lipid peroxidation (Li et al., 2019a). These results provide evidence that treatment with mefunidone partially inhibited renal oxidative stress.

Apoptosis is a type of programmed cell death that maintains the physiological function of organs to a certain extent by removing non-functional or damaged cells. However, excessive apoptosis adversely affects the body (Priante et al., 2019). Apoptosis of renal tubular epithelial cells is an important mechanism in IRI-induced nephropathy (Li et al., 2021b; Kim et al., 2021; Rahbar Saadat et al., 2021). Here, we revealed that mefunidone treatment significantly suppressed renal apoptosis induced by IRI using TUNEL staining and concluded that mefunidone can reduce the expression of the apoptotic marker cleaved-caspase3. Subsequently, we detected apoptosis by flow cytometry and cleaved-caspase3 protein expression by western blotting *in vitro*, and the results were consistent with those *in vivo*. This suggests that mefunidone alleviates renal injury by inhibiting renal apoptosis.

Mitochondrial dysfunction is an important factor that affects the progression of AKI (Clark and Parikh, 2020). The mitochondrial pathway is also the main pathway for apoptosis (Dai et al., 2017; Li et al., 2019b; Carneiro and El-Deiry, 2020). First, we observed morphological changes in the renal mitochondria using transmission electron microscopy, and the results showed that mefunidone has a distinct protective action against IRI-induced mitochondrial damage by reducing mitochondrial swelling and vacuolation. The bcl2 and caspase families are key molecules in regulating mitochondrial pathway apoptosis. The pro-apoptotic protein Bax promotes the release of apoptosis factors by increasing the permeability of the mitochondrial outer membrane and ultimately upregulates the protein level of apoptosis marker cleaved-caspase3 to induce apoptosis, while anti-apoptotic protein bcl2 inhibits the above process (Chen et al., 2019b; Wang et al., 2020). Thus, we further explored whether mefunidone alleviates renal injury by protecting the mitochondria and regulating the expression of mitochondrial-related apoptotic proteins. Bax and bcl2 expression was tested by western blotting, and the expression of Bax was upregulated while the expression of bcl2 was downregulated in the IRI group, and the expression of the above proteins was partially reversed by treatment with mefunidone. The detection of protein expression in the mitochondrial electron transport chain also revealed that mefunidone could improve the mitochondrial electron transport chain complex III and complex V levels. These conclusions were verified both *in vivo* and *in vitro*. In addition, we observed that mefunidone maintained MMP in HK-2 cells. Based on these results, we conclude that mefunidone ameliorated IRI-induced AKI, at least in part, by reducing renal apoptosis in the mitochondrial pathway.

High doses of FA-induced AKI, which is also a classic AKI model, simulate drug- or toxin-induced tubular injury. High doses of FA can cause direct toxicity to renal tubular epithelial cells or block renal tubules by forming crystals in the renal lumen (Martin-Sanchez et al., 2017; Martin-Sanchez et al., 2018; Wang et al., 2021). High doses of FA are applied in the treatment of certain gastrointestinal cancers, which increase the incidence of high-dose FA-induced renal injury (Metz-Kurschel et al., 1990; Li et al., 2020). Therefore, we conducted an efficacy experiment with mefunidone on FA-induced AKI. We found that mefunidone significantly reduced pathological damage to renal tubules, improved renal function, decreased NGAL expression, and inhibited apoptosis.

In this study, we investigated the role of mefunidone in IRI and FA-induced AKI. Mefunidone ameliorated the oxidative stress, mitochondrial damage, and renal apoptosis induced by IRI. And through our observations, mefunidone reduced renal fibrosis in post-AKI develop CKD. It means that renal damage can be mitigated by prophylactic treatment with mefunidone before it occurs. In addition, mefunidone had a significant effect on FA-induced AKI. Therefore, we concluded that mefunidone has the potential to protect against AKI.

However, as a small-molecular-weight compound, its specific therapeutic targets remain unclear, and exploring the specific target of mefunidone is still encountering great technical challenges. This aspect deserves further exploration in the future.

## Data Availability

The original contributions presented in the study are included in the article/Supplementary Material, further inquiries can be directed to the corresponding author.

## Supplementary material

SUPPLEMENTARY FIGURE S1 Mefunidone alleviated kidney fibrosis and inhibited EMT in IRI-induced CKD. **(A)** HE staining (×200) and tubular injury scores showed protective effect of mefunidone on renal tubular injury on day 14 after IRI modeling. arrows for renal tubular damage. **(B)** Masson’s trichrome staining (×200) and kidney fibrotic scores showed protective effect of mefunidone on renal tubular injury on day 14 after IRI modeling. arrows for renal tubular damage and renal fibrosis. **(C)** Histological images of immunohistochemical staining with collagen I and evaluation of collagen I positive area in each group on day 14 after IRI modeling (×200). **(D)** Histological images of immunohistochemical staining with vimentin and evaluation of vimentin positive area in each group on day 14 after IRI modeling (×200). **(E)** Western blot analysis and quantitative data of α-SMA and E-Cadherin in each group on day 14 after IRI modeling. Mefunidone: 100 mg/kg. Data represent mean ± SEM (n = 5–6). **p* < 0.05, vs Sham group; ***p* < 0.01, vs Sham group; ****p* < 0.001, vs Sham group; *****p* < 0.0001, vs Sham group; ^#^ *p* < 0.05, vs IRI group; ^##^ *p* < 0.01, vs IRI group; ^###^ *p* < 0.001, vs IRI group.

SUPPLEMENTARY FIGURE S2 CCK-8 was used to explore the optimal drug concentration of mefunidone on HK-2 cells viability. HK-2 cells were treated with mefunidone at concentrations of 0, 20, 40, 80, 120 and 160 µg/ml. **p* < 0.05, ***p* < 0.01.
