# Corrigendum: Protective effects of Mefunidone on ischemia-reperfusion injury/folic acid-induced acute kidney injury

**DOI:** 10.3389/fphar.2023.1188615

**Published:** 2023-04-05

**Authors:** Jiajia Li, Yupeng Jiang, Qin Dai, Yue Yu, Xin Lv, Yan Zhang, Xiaohua Liao, Liyun Ao, Gaoyun Hu, Jie Meng, Zhangzhe Peng, Lijian Tao, Yanyun Xie

**Affiliations:** ^1^ Department of Nephrology, Xiangya Hospital, Central South University, Changsha, China; ^2^ Hunan Key Lab of Organ Fibrosis, Changsha, China; ^3^ National International Collaborative Research Center for Medical Metabolomics, Xiangya Hospital, Central South University, Changsha, China; ^4^ Department of Oncology, The Second Xiangya Hospital, Central South University, Changsha, China; ^5^ Department of Medicinal Chemistry, Xiangya School of Pharmaceutical Sciences, Central South University, Changsha, China; ^6^ Department of Pulmonary and Critical Care Medicine, The Third Xiangya Hospital, Central South University, Changsha, China

**Keywords:** renal ischemia-reperfusion injury, folic acid, acute kidney injury, chronic kidney disease, mefunidone

In the published article, there was an error in the legend for Figure 1F as published. Figure 1F was displayed as “Vimentin positive area (%).” The correct Figure 1F is “NGAL positive area (%).” The corrected legend appears below.

**FIGURE 1:**
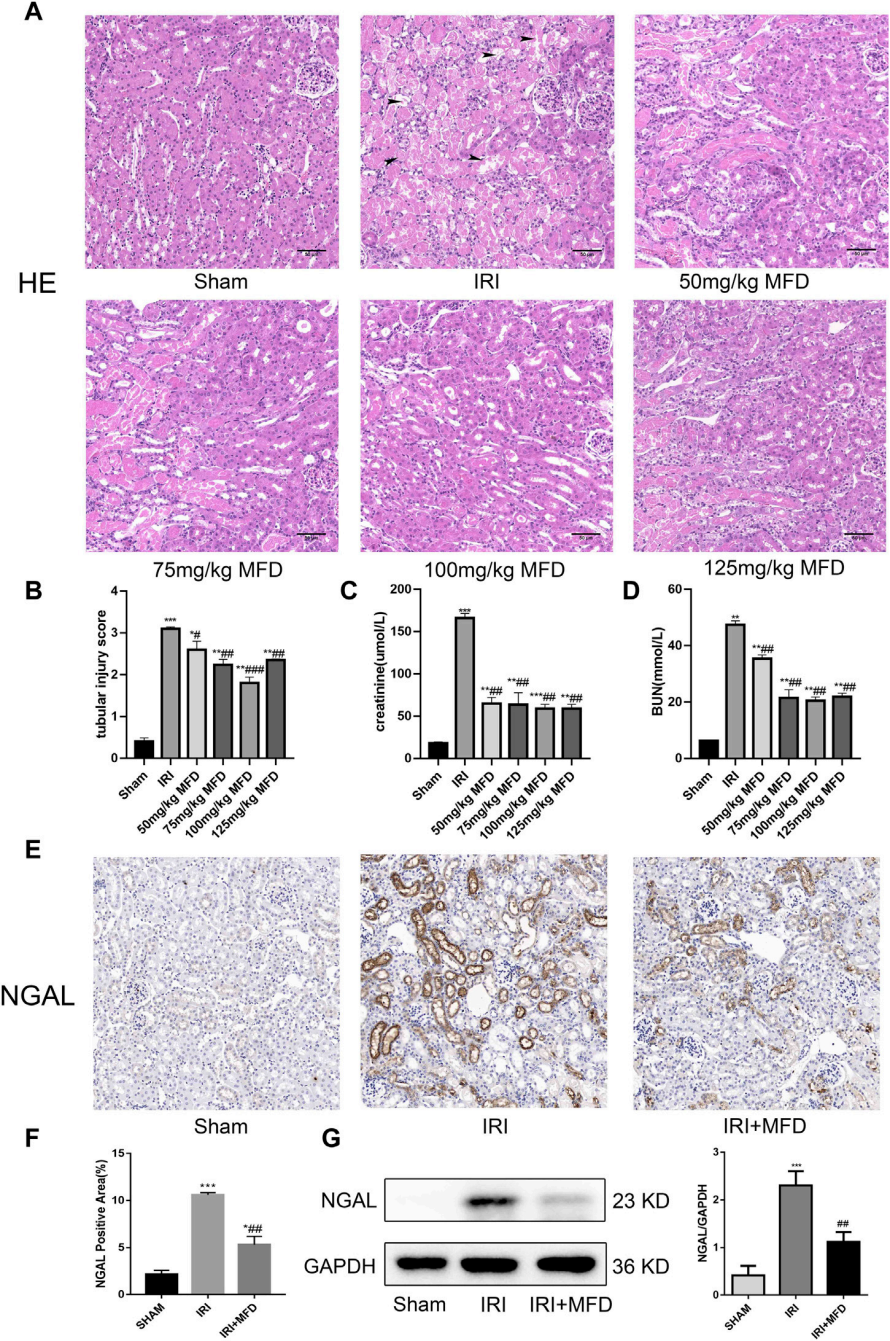
Mefunidone ameliorated IRI-induced AKI **(A)** HE staining showed protective effect of mefunidone at various doses of 50 mg/kg, 75 mg/kg, 100 mg/kg, 125 mg/kg on renal tubular injury on day 2 after IRI modeling (×200). arrows for renal tubular damage. **(B)** The tubular injury scores of HE staining for kidney damage. **(C)** Serum creatinine (SCr) levels of mefunidone at various doses of 50 mg/kg, 75 mg/kg, 100 mg/kg, 125 mg/kg on renal tubular injury on day 2 after IRI modeling. **(D)** Blood urea nitrogen (BUN) levels of mefunidone at various doses of 50 mg/kg, 75 mg/kg, 100 mg/kg, 125 mg/kg on renal tubular injury on day 2 after IRI modeling. **(E,F)** Histological images of immunohistochemical staining with NGAL and evaluation of NGAL positive area in each group on day 2 after IRI modeling (×200). Mefunidone: 100 mg/kg. **(G)** Western blot analysis and quantitative data of NGAL in each group on day 2 after IRI modeling. Mefunidone: 100 mg/kg. Data represent mean ± SEM (*n* = 3–5). *p < 0.05, vs. Sham group; **p < 0.01, vs. Sham group; ***p < 0.001, vs. Sham group; #*p* < 0.05, vs. IRI group; ##*p* < 0.01, vs. IRI group; ###*p* < 0.001, vs. IRI group.

Furthermore, there was an error in the **Supplementary Material**. **Supplementary Figure S1** was displayed as “CCK-8 to determine the optimal drug concentration of mefunidone for 24 h for HK-2 cell viability”; **Supplementary Figure S2** was displayed as “Mefunidone alleviated kidney fibrosis and inhibited EMT in IRI-induced CKD.” The correct **Supplementary Figure S1** is “Mefunidone alleviated kidney fibrosis and inhibited EMT in IRI-induced CKD”; The correct **Supplementary Figure S2** is “CCK-8 to determine the optimal drug concentration of mefunidone for 24 h for HK-2 cell viability.”

The authors apologize for this error and state that this does not change the scientific conclusions of the article in any way. The original article has been updated.

## Supplementary material

SUPPLEMENTARY FIGURE S1 Mefunidone alleviated kidney fibrosis and inhibited EMT in IRI-induced CKD **(A)** HE staining (×200) and tubular injury scores showed protective effect of Mefunidone on renal tubular injury on day 14 after IRI modeling. arrows for renal tubular damage. **(B)** Masson's trichrome staining (×200) and kidney fibrotic scores showed protective effect of Mefunidone on renal tubular injury on day 14 after IRI modeling. arrows for renal tubular damage and renal fibrosis. **(C)** Histological images of immunohistochemical staining with collagen I and evaluation of collagen I positive area in each group on day 14 after IRI modeling (×200). **(D)** Histological images of immunohistochemical staining with vimentin and evaluation of vimentin positive area in each group on day 14 after IRI modeling (×200). **(E)** Western blot analysis and quantitative data of α-SMA and E-Cadherin in each group on day 14 after IRI modeling. Mefunidone: 100 mg/kg. Data represent mean ± SEM (n = 5-6). **p* < 0.05, vs Sham group; ***p* < 0.01, vs Sham group; ****p* < 0.001, vs Sham group; *****p* < 0.0001, vs Sham group; #*p* < 0.05, vs IRI group; ##*p* < 0.01, vs IRI group; ###*p* < 0.001, vs IRI group.

SUPPLEMENTARY FIGURE S2 CCK-8 to determine the optimal drug concentration of Mefunidone for 24 h for HK-2 cell viability. HK-2 cells were treated with Mefunidone at concentrations of 0, 20, 40, 80, 120 and 160 μg/ml. **p* < 0.05, ***p* < 0.01.

