# Supplementary figures and images for: Protective effects of mefunidone on ischemia-reperfusion injury/Folic acid-induced acute kidney injury
Source: Front Pharmacol. 2022 Nov 23;13:1043945. doi: 10.3389/fphar.2022.1043945 (PMC9727196; doi:10.3389/fphar.2022.1043945)

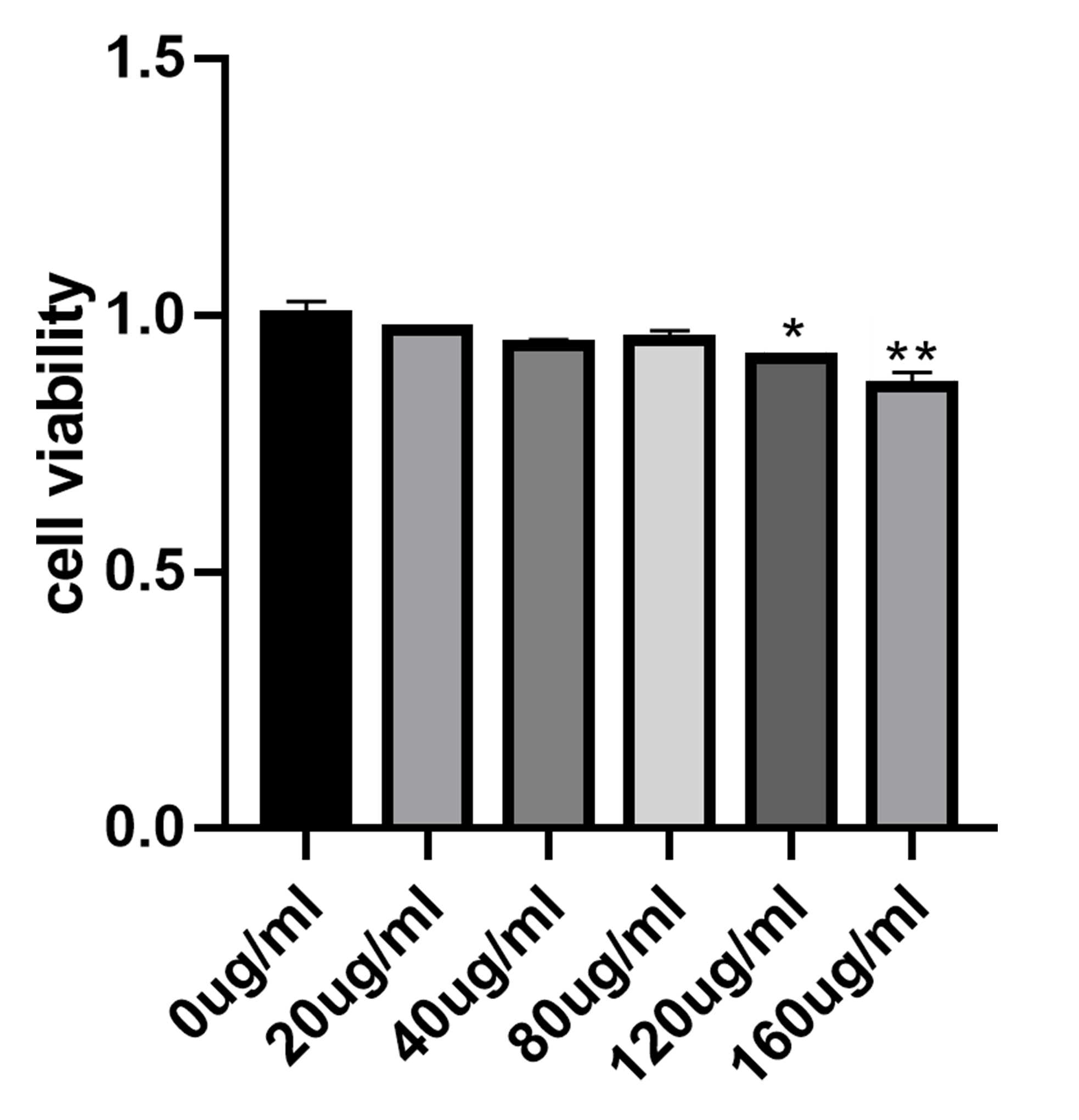

Supplement: Supplementary file 1 [file Image2.tif]
